# A prospective association between dietary mushroom intake and the risk of type 2 diabetes: the Korean Genome and Epidemiology Study–Cardiovascular Disease Association Study

**DOI:** 10.4178/epih.e2024017

**Published:** 2024-01-08

**Authors:** Yu-Mi Kim, Hye Won Woo, Min-Ho Shin, Sang Baek Koh, Hyeon Chang Kim, Mi Kyung Kim

**Affiliations:** 1 Department of Preventive Medicine, Hanyang University College of Medicine, Seoul, Korea; 2 Institute for Health and Society, Hanyang University, Seoul, Korea; 3 Department of Preventive Medicine, Chonnam National University Medical School, Gwangju, Korea; 4 Department of Preventive Medicine and Institute of Occupational Medicine, Yonsei Wonju College of Medicine, Wonju, Korea; 5 Department of Preventive Medicine, Yonsei University College of Medicine, Seoul, Korea

**Keywords:** Dietary mushrooms, Prospective studies, Risk, Type 2 diabetes mellitus, Republic of Korea

## Abstract

**OBJECTIVES:**

Mushrooms, known for their nutritious and functional components, are considered healthy and medicinal. This study investigated the prospective association between dietary mushroom consumption and the incidence of type 2 diabetes among Korean adults aged ≥40 years.

**METHODS:**

In total, 16,666 participants who were not taking anti-diabetic medication or insulin and had normal fasting blood glucose (FBG; <126 mg/dL) were included. We used the cumulative average dietary consumption of mushrooms as an exposure metric, calculated from food frequency questionnaires at every follow-up, along with covariates collected during a baseline survey. To estimate incidence rate ratios (IRRs) for type 2 diabetes, a modified Poisson regression model with a robust error estimator was applied.

**RESULTS:**

In multivariable models, dietary mushroom consumption was inversely associated with type 2 diabetes incidence in both genders (men: IRR, 0.65; 95% confidence interval [CI], 0.47 to 0.90; p_linearity_=0.043 in the highest quartile (Q4) vs. the lowest quartile (Q1); women: IRR, 0.70; 95% CI, 0.54 to 0.93; p_linearity_=0.114 in Q4 vs. Q1). The inverse association remained after adjustment for dietary factors instead of dietary quality index, the baseline FBG, and the exclusion of incidence within the first year. Additionally, no significant interaction was found regarding the risk of type 2 diabetes between dietary mushroom consumption and participants’ gender or other factors.

**CONCLUSIONS:**

Dietary mushroom consumption was inversely linked with the risk of type 2 diabetes incidence in both genders, indicating the beneficial role of mushrooms in preventing the disease.

## GRAPHICAL ABSTRACT

[Fig f1-epih-46-e2024017]

## Key Message

• Increased dietary consumption of commonly used mushrooms is associated with a lower risk of type 2 diabetes incidence among adults aged 40 years or older in Korea.

• This inverse relationship remains consistent across genders and various dietary backgrounds, underscoring the potential of mushrooms as a preventive dietary component against.

## INTRODUCTION

In 2019, type 2 diabetes (T2D) accounted for 2.61% of the disability-adjusted life-years (DALYs) worldwide, a burden that has increased 1.66 times from 0.98% in 1990 (an increase of 380.63 DALYs per 100,000) [1]. According to an estimate in 2017, 6.28% of the world’s population suffers from T2D, with annual deaths reaching 1 million, making it the ninth leading cause of death [2]. From 2012 to 2020, a notable increase occurred in the age-standardized prevalence of T2D in Korea, with rates rising from 12.4% to 19.2% in men and from 11.1% to 14.3% in women aged ≥ 30 years [3]. Accordingly, the prevention of T2D has become a major public health priority. Numerous studies have been conducted on its modifiable risk factors, such as insufficient physical activity and dietary factors, and its non-modifiable risk factors, such as age and specific genetic or family history [4]. In addition, lifestyle modification can be a key factor in reducing the risk of T2D and slowing its progression with minimal cost [5].

The associations between dietary factors and the incidence of T2D have been studied extensively. Strong evidence supports both positive and inverse relationships, with a range of dietary components. For instance, the consumption of whole grains and cereal fibre is linked to lower risk, whereas moderate alcohol consumption, red meat, processed meat, bacon, and sugar-sweetened beverages are well-established risk factors [5,6]. Nevertheless, further studies are needed to obtain high-quality evidence and identify novel dietary factors that may help prevent T2D.

Mushrooms and their extracts have been proposed to have a beneficial influence on health conditions against oxidative stress [7], inflammation [8], and atherogenic lipid profile [9] through in vitro and animal studies. They are generally considered a healthy food because they are good sources of vitamins (B1, B2, B12, biotin, folate, C, and D), essential minerals (iron, magnesium, phosphorus, potassium, zinc, and selenium), and bioactive compounds such as beta-glucan, ergosterol, ergothioneine, and polysaccharides [10]. However, few human studies have focused on the association between mushroom consumption and diabetes or blood glucose, and they had limitations such as small sample sizes [11] and cross-sectional design [12]. A recent prospective study, which combined the Nurses’ Health Study and the Health Professional Follow-up Study, evaluated the association between mushroom consumption and T2D, but a significant association was not found [13]. The study pointed out that it would be necessary to examine the possibility in other populations with different cultural norms, in which mushrooms are commonly eaten [13].

According to the Food and Agriculture Organisation of the United Nations, in 2020 the worldwide production of mushrooms and truffles totalled approximately 42.9 million tons, mostly in Asia (95.4%), followed by Europe (3.3%) [14]. In Korea, mushrooms are commonly used as side dishes or medicinal material, and the annual consumption as a raw material in the food industry is estimated to be 735 tons, a large amount when compared internationally [15]. Therefore, the present study aimed to examine whether the dietary consumption of commonly used mushrooms has an inverse association with the occurrence of T2D based on a prospective cohort study among men and women aged ≥ 40 years in Korea.

## MATERIALS AND METHODS

### Genome and Epidemiology Study–Cardiovascular Disease Association Study cohort and analytic population

The Korean Genome and Epidemiology Study–Cardiovascular Disease Association Study (KoGES_CAVAS) is a combined study of 3 community cohorts: the Multi-Rural Communities Cohort (MRCohort) of 3 rural areas, namely Yangpyeong, Namwon, and Goryeong; the Atherosclerosis Risk of Rural Areas in the Korean General Population (ARIRANG) cohort of 2 rural areas, Wonju and Pyeongchang; and the Kangwha cohort. It was established to identify risk factors comprehensively among Koreans for cardiometabolic conditions, including T2D [16]. Since 2005, these 3 cohorts covering a total of 6 counties have participated in the KoGES. Each cohort enrolled community-dwellers aged ≥ 40 years using multistage cluster sampling between January 2005 and December 2011 (9,759 participants from the MRCohort, 5,942 participants from the ARIRANG cohort, and 3,845 participants from the Kangwha cohort). A total of 19,546 participants without cardiovascular disease or cancer were followed-up every 2-4 years between 2007 and 2017, with 78.2% having more than 1 follow-up visit (median period between visits, 2.3-3.5 years).

We excluded individuals with the following conditions at the baseline survey: having a prior diagnosis of T2D with anti-diabetic medication or insulin use, having a fasting blood glucose (FBG) level ≥ 126 mg/dL (n = 2,156), having ≥ 10 items blank on the food frequency questionnaire (FFQ) or an implausible energy intake (defined as values ≤ 0.5th or ≥ 99.5th percentiles of total energy intake, with cut-offs of ≤ 619 or ≥ 4,032 kcal/day in men and ≤ 509 or ≥ 3,918 kcal/day in women; n= 284), or missing data on covariates including educational level, regular exercise, smoking status, and alcohol consumption (n= 440). As a result, 16,666 participants (6,162 men and 10,504 women) were included in the final analysis.

To overcome the limitations of a multicentre study, the same personnel from the coordinating centre trained interviewers and measurers with videos, field inquiry, and hands-on exercises, and comprehensive health examinations were conducted using standard protocols.

### Assessment of dietary exposure and dietary covariates

At baseline and each follow-up visit (2-4 year intervals), trained interviewers collected dietary data using a validated FFQ with 106 items [16]. Participants were asked how frequently they consumed each food item and what its average portion size had been during the preceding year. Food photographs with portion sizes were provided to improve the reliability of participants’ answers. The nine options for frequency ranged from “never or rarely” to “3 times per day,” and three portion sizes were specified for each item [17]. Mushroom intake was assessed using 2 food categories: (1) *Pleurotus ostreatus* (oyster mushroom) and (2) other mushrooms, including *Auricularia heimuer* (wood ear mushroom), *Agaricus bisporus* (button mushroom), *Flammulina velutipes* (winter mushroom), and *Lentinula edodes* (oak mushroom). The standard portion size was 30 g of raw mushroom. The cumulative daily mushroom intake (serving/day) was calculated by averaging the mushroom intake at baseline and each follow-up survey before the endpoint, or censoring to reduce within-participant variation and reflect long-term dietary mushroom intake [18]. The average FFQ value used to estimate the cumulative average consumption of dietary factors was 2.04, with a range between 1 and 3.

Nutrient intakes were calculated using weighted frequencies per day and serving sizes per unit for each food item from the 2011 nutrient database of the Korean Nutrition Society [19], which is based on the seventh edition of the Korean Food Composition Table [20]. The modified Diet Quality Index-International (DQI-I) score, used as a dietary covariate to assess dietary quality, was calculated based on the following foods and nutrients: vegetables, fruits, grains, fibre, protein, iron, calcium, vitamin C, total fats, saturated fats, cholesterol, sodium, the ratio of carbohydrates to proteins to fats, and the ratio of fatty acids (polyunsaturated fatty acid to monounsaturated fatty acid to saturated fatty acid) [21]. The scoring criteria are provided in Supplementary Material 1. The DQI-I score ranges from 0 to 100, and a higher score indicates better dietary quality. All nutrients and the DQI-I score were also used as averaged values.

### Ascertainment of diabetes incidence

At every follow-up examination, the participants were asked whether they had been diagnosed with T2D and treated with any anti-diabetic medications or insulin. Blood samples were collected in the morning after at least 8 hours of fasting. In addition, each participant’s FBG level was measured using an ADVIA1650 Automatic Analyser (Siemens, New York, NY, USA). We defined the incidence case of T2D with these conditions at follow-up: (1) self-report of T2D newly diagnosed by a physician, such that the participant had started taking anti-diabetic medications or insulin, or (2) elevated level of FBG ≥ 126 mg/dL during a follow-up visit. This definition is primarily derived from the criteria of the Korean Diabetes Association [22]. However, we refined the first condition to include only those patients who had begun treatment with an anti-diabetic medication or insulin. This adjustment was made for 2 reasons: first, we did not have access to medical records confirming newly diagnosed T2D; second, it was deemed necessary to address the potential for subjective interpretation in self-reported diagnoses. Additionally, we were able to use blood tests to identify patients with T2D not revealed through self-reporting.

### Assessment of non-dietary covariates

Data on age, gender, educational level, regular exercise, smoking status, drinking status, and alcohol consumption were collected by trained interviewers using a structured questionnaire. The following definitions were used in the analyses: high school graduate, 12 years of schooling or more; and regular exercise, ≥ 3 times/wk and ≥ 30 min/session. In addition, we calculated total alcohol consumption (g/day) by multiplying the total volume of all alcoholic beverages consumed in the previous year, based on the average frequency and the number of typical glasses of alcoholic beverages commonly consumed in Korea, by the specific gravity of alcohol (0.795).

Height was measured using a stadiometer to the nearest 0.1 cm, and weight was assessed with a metric scale to the nearest 0.1 kg with participants wearing light clothing and no shoes. We calculated body mass index (BMI) using the ratio of weight (kg) to height squared (m^2^).

### Statistical analysis

Participants were categorised into quartiles according to their average intake of dietary mushrooms. The baseline characteristics were represented as means with standard deviation (SD) for continuous variables or frequencies with percentages for categorical variables. A general linear model was used to present both age-adjusted estimates and linear trends for general characteristics according to the participants’ quartiles of cumulative average dietary consumption of mushrooms. The linear trends were tested by treating the median value of each quartile as a continuous variable. We examined the potentially non-linear relation between dietary mushroom intake and the risk of T2D non-parametrically with restricted cubic splines [23]. To check for non-linearity, we used the likelihood ratio test, comparing the model with only the linear term to the model with the linear and cubic spline terms.

To estimate incidence rate ratios (IRRs) and 95% confidence intervals (CIs), we used a modified Poisson regression model with a robust error estimator [24,25]. The person-time of follow-up visits for each participant was calculated from the date of enrolment to the date of self-reported diagnosis of T2D with treatment (medication or insulin), diagnosis through blood tests at a follow-up visit, or the end of follow-up between 2014 and 2017, whichever came first. When participants were lost to follow-up, half the median follow-up time of the participants who were successfully followed up was assigned as their follow-up time [26]. We did not consider the date of death, treating these participants the same as those lost to follow-up. Three models were presented: (1) age-adjusted; (2) multivariable-adjusted model (age [years], educational level [≥ 12 years, yes or no], regular exercise, smoking status [non-smoker, former, or current], alcohol consumption [mL/day], BMI [kg/m^2^], and total energy intake [kcal/day]) [5,6]; and (3) a model that aimed to reflect the common belief that mushrooms are nutritious and healthy, implying that individuals who prefer mushrooms would likely have healthy dietary habits [27]; thus, the modified DQI-I score [21] was included as a potential confounder in our final model [28]. For these covariates, except for dietary factors, the values obtained during the baseline survey were used.

To test the robustness of our findings, we performed sensitivity analyses. First, we evaluated the influence of adjustment for nutrients related to diabetes, including dietary calcium (mg/day), folate (μg/day), isoflavones (mg/day), glycaemic load, iron (mg/day), fibre (g/day), and magnesium (mg/day) [5,6,29,30], instead of the modified DQI-I score. All nutrient intake values in the sensitivity analysis were total energy-adjusted values using the residual method [31]. Second, we adjusted for the baseline FBG value. Third, to account for the possibility of reverse causality bias, we repeated all analyses after excluding T2D events that occurred within the first year.

To assess potential interactions between T2D risk factors and dietary mushroom intake, we conducted stratified analyses by age (< 65 compared to ≥ 65 years), educational level (< 12 compared to ≥ 12 years), regular exercise (yes or no), smoking and drinking status (never/past compared to current), BMI (< 23 or ≥ 23 kg/m^2^), prediabetic status (FBG < 100 compared to ≥ 100 mg/dL at baseline), and the modified DQI-I score (below the median compared to at or above the median). We tested their interactions using a cross-product term in a Poisson regression.

The analyses were carried out using SAS version 9.4 (SAS Institute Inc., Cary, NC, USA), and the level of significance was set at 0.05. However, for stratification analysis and interaction analysis, p-values were adjusted for multiple testing using the Bonferroni correction, with a corrected significance level of < 0.05/16= 0.0031 for the stratification analysis and < 0.05/8= 0.0063 for the interaction analysis in both men and women.

### Ethics statement

The study protocol was approved by the Institutional Review Boards of Hanyang University (HYG-07-01), Chonnam National University (06-062), Keimyung University (DSMC 2013-05-016-001), Yonsei Wonju College of Medicine (CR105024), and Yonsei University (4-2010-0272). All participants provided written informed consent prior to this study.

## RESULTS

During the follow-up period (median, 5.98 years; interquartile range, 3.23 to 8.77), a total of 96,945 person-years of follow-up were accumulated in the CAVAS (35,831 person-years for men and 61,114 for women; 50,287 in the MRCohort, 29,442 in the ARIRANG cohort, and 17,216 in the Kangwha cohort), and 945 incident cases of T2D occurred (410 cases in men and 535 in women; 533 in the MRCohort, 252 in the ARIRANG cohort, and 160 in the Kangwha cohort). The age-adjusted baseline characteristics according to quartiles of the cumulative average mushroom intake are presented in Table 1. The men and women in the highest quartiles of mushroom intake were likely to be younger, highly educated, regular exercisers, and non-smokers, with higher total energy intake and quality index scores for both men and women. In addition, men in the highest quartile were likely to have a higher BMI, and those in the lowest quartile tended to drink more alcohol, but no tendency was identified across quartiles for women. As for adjusted nutrients instead of the modified DQI-I score, the men and women in the highest quartile tended to have higher levels of intake of calcium, folate, isoflavones, iron, fibre, and magnesium (Supplementary Material 2). Additionally, they had a lower glycaemic load.

The multivariable-adjusted IRRs (95% CIs) for T2D incidence across quartiles of mushroom intake are shown in Table 2. After adjustment for established risk factors of T2D, we found a significant inverse association with the risk of T2D. Although the additional adjustment for the modified DQI-I score did not substantially alter the association, the inverse associations were robust in both men and women (IRR for the highest vs. lowest quartile in the model including the modified DQI-I score, for men: IRR, 0.65; 95% CI, 0.47 to 0.90; p_linearity_= 0.043; for women: IRR, 0.70; 95% CI, 0.54 to 0.93; p_linearity_= 0.114). Adjusting for the 7 nutrients instead of the DQI-I did not substantially alter the associations. However, notably, while a linear trend was found in men, the association in women appeared to be significantly non-linear, following an L-shaped pattern (p_non-linearity_= 0.027). These trends remained in the sensitivity analysis that included the 7 nutrients. In the sensitivity analysis among non-users of multi-nutrients and functional food supplements, the non-linearity in women disappeared (p_non-linearity_= 0.145). When we adjusted for FBG at baseline, excluded cases within the first year of follow-up, and excluded users of multi-nutrients or functional food supplements, the inverse associations remained robust.

In the stratified analyses, the associations between mushroom consumption and the risk of T2D persisted in all subgroups, and no significant effect modification was observed for mushroom consumption and age, educational level, regular exercise, smoking status, drinking status, BMI, prediabetes condition, or modified DQI-I score (all p-values for interactions ≥ 0.003; the significance levels of the Bonferroni correction for 16 multiple tests are shown in Table 3).

## DISCUSSION

The present prospective cohort study with 16,666 participants and 96,945 person-years demonstrated an inverse association between the average dietary consumption of mushrooms and the risk of T2D. Consumption of mushrooms was likely slightly higher in women than in men. However, significant inverse associations appeared for both men and women, and the tendencies were similar in men and women. In the stratified analyses by each covariate, no significant effect modification was found.

In the multivariable-adjusted model, which included non-dietary and dietary potential confounders, we found lower IRRs by approximately 24-35% in the highest quartile (Q4) compared to the lowest quartile (Q1). These inverse associations remained robust even after we considered the baseline FBG or excluded those who developed T2D in the first year of follow-up. Unfortunately, little research has been published previously on the association between mushroom consumption and the risk of T2D. The only prospective study conducted on men and women in the United States [13] could not detect a beneficial association for mushroom consumption but found a possible inverse association between T2D and consuming mushrooms instead of processed meat, poultry, or fish. They suggested that the inverse association of substitution may reflect a reduction in T2D risk due to foods such as processed meat, which was reported to have a positive association with the risk of T2D [32], rather than the absolute beneficial effect of mushrooms. However, in the present study, the average amount of unprocessed red meat or processed red meat consumption was relatively low (41.9 and 0.95 g/day for men and 22.8 and 0.97 g/day for women, respectively). Considering red meat, processed meat, and both as additional confounders did not change their associations (data not shown). Besides, a previous cross-sectional analysis of mushroom consumption was conducted regarding FBG and the prevalence of T2D in Italian adults without diabetes; however, they showed positive associations between mushroom consumption and FBG (8.46 mg/dL increase per 30 g/wk increment) [12]. This unexpected positive association may be because of reverse causation. Recently, a prospective cohort analysis using the U.S. National Health and Nutrition Examination Survey (NHANES) III reported that mushroom consumption was inversely associated with all-cause mortality (hazard ratio, 0.32; 95% CI, 0.06 to 1.65), but it was not associated with diabetes mellitus mortality [33].

We could not clearly explain this discrepancy from our beneficial findings on dietary mushroom consumption; however, it may be related to the absolute amounts or the types of mushrooms consumed. First, as we mentioned above, in Korea, mushrooms are commonly used as side dishes or medicinal material, and the total amount used annually in Korea is quite large when compared internationally [15]. The average dietary intake of mushrooms reported in the Korean NHANES was stable at approximately 4-5 g/day from 2000 to 2009; subsequently, it increased from 4.4 g/day in 2009 to 7.3 g/day in 2019 among adults aged ≥ 19 years in Korea [34]. In the present study, the average daily intake of mushrooms was 0.11 serving/day (3.39 g/day) and 0.14 serving/day (4.32 g/day) in men and women, respectively, showing right-skewed distributions; the median values in the fourth quartile, 0.25 serving/day for men (7.5 g/day) and 0.30 serving/day for women (9.0 g/day), have been reported. However, these values could be underestimated, considering a Korean dietary culture in which mushrooms, especially *Lentinula edodes* (oak mushroom), are often used to prepare broth and ingredients for various side dishes; thus, our study’s participants may not have been aware of how much mushroom was used in their meals. Further studies examining dietary mushroom consumption in detail are needed to confirm the present study’s findings, although the amount of mushroom consumption may vary according to individual preferences. Second, the mushrooms most commonly used in the United States are variants of the cultivated mushroom (button, cremini, and portabella) [35], whereas oyster, winter, oak, button, and wood ear mushrooms were selected in the present study [17]. This difference in commonly used types of mushrooms between the two study populations may partly explain the discrepancy between them. However, because we found no evidence of an association specific to mushroom type, we analysed two mushroom categories for T2D risk. Separately, the significant inverse associations remained when oyster mushrooms and all other mushrooms (winter, oak, button, and wood ear) were considered (data not shown).

Mushrooms contain various compounds, such as polysaccharides and phytochemicals, that are biochemically active against oxidative stress [10,27,35,36]. Among mushrooms commonly eaten by the present study population, the dietary fibre fraction of *Pleurotus ostreatus* (oyster mushroom) was reported to potentially lower blood cholesterol [36,37]. Little evidence exists for each mushroom species or its components regarding the underlying mechanisms in the development of T2D. However, considering the low calorie-to-weight ratio and high fibre content of mushrooms, the observed association is likely to be related to the effects of fibre and low glycaemic load [38], which are known to reduce the occurrence of T2D. Even after adjusting for glycaemic load and fibre (in the multivariable model with nutrients instead of the DQI-I), significant inverse associations remained. Mushrooms, including those commonly consumed in Korea (especially winter, button, and oak mushrooms), are abundant in folic acid [39], vitamin D [40], and zinc [41], which are known to reduce the risk of T2D. Furthermore, an in vitro study found that they contained phenolic compounds (flavonoids) and potentially had anti-oxidative effects [42]. Besides micronutrients and bioactive compounds that have potential beneficial properties against the development of T2D, the dietary intake of selenium, which is an essential constituent of some enzymes with antioxidant functions and which mushrooms can supply, is also considered to affect the risk of T2D [43].

The overall tendency in men and women was very similar, and no gender-based difference was found in the present study (p_interaction_= 0.659). However, a significant linear trend was observed for men but not women, whereas in the final model, a non-linear trend of inverse association was found for women (p_non-linearity_= 0.027). This may be related to the unmeasured use of medicinal mushrooms, such as homemade medicinal mushroom extracts from *Ganoderma lucidum* or *Phellinus linteus*. Thus, while we did not directly account for the medicinal use of mushrooms, we conducted a sensitivity analysis after excluding users of functional food and multi-nutrient supplements as a surrogate variable to address this issue. We observed that despite the exclusion of approximately 50% of the cases and person-years (as shown in Supplementary Material 2), the inverse associations remained significant in the highest quartiles for both men and women (Table 2). Furthermore, the non-linearity in women disappeared. As mentioned earlier, while we could not completely rule out the influence of the medicinal use of mushrooms, the results of this sensitivity analysis support the potential preventive benefits of dietary mushroom consumption against the development of T2D.

Several limitations may have affected the interpretation of our findings. First, a strength of the present study is that the cumulative average mushroom intake was calculated from repeated assessments during follow-up to reflect long-term mushroom consumption and reduce the measurement errors possible from a single sampling [18]. Second, as we mentioned before, estimating dietary mushroom intake from only two food categories, including “other mushrooms,” may have influenced participants’ perception of mushroom types and led them potentially to mischaracterise their mushroom consumption. In addition, the possibility remains that the use of medicinal mushrooms, which we did not measure, may obscure the true association [44]. In further research on mushrooms, it will be necessary to focus on foods related to T2D risk and use medicinal mushroom intake as a covariate. Third, the questionnaire and health examination were assessed using a standard protocol provided for the KoGES. However, the KoGES_CAVAS itself is also a multicentre cohort; therefore, we conducted additional cohort-stratified analyses and then pooled their results (Supplementary Material 3). Similar associations were found in all multivariable models (for men in the multivariable model with DQI-I: pooled IRR, 0.63 in Q4 vs. Q1; 95% CI, 0.46 to 0.88; p_linearity_= 0.676; for women: pooled IRR, 0.70; 95% CI, 0.52 to 0.93; p_linearity_= 0.087).

In conclusion, the present study’s findings indicate that the average consumption of dietary mushrooms may be beneficial for preventing the development of T2D. However, future large-scale prospective studies with a broader scope, including the use of medicinal mushrooms or considering it as a covariate, are needed to confirm and determine the specific impact of mushroom consumption on the risk of T2D and its dose-response relationship.

## Figures and Tables

**Figure f1-epih-46-e2024017:**
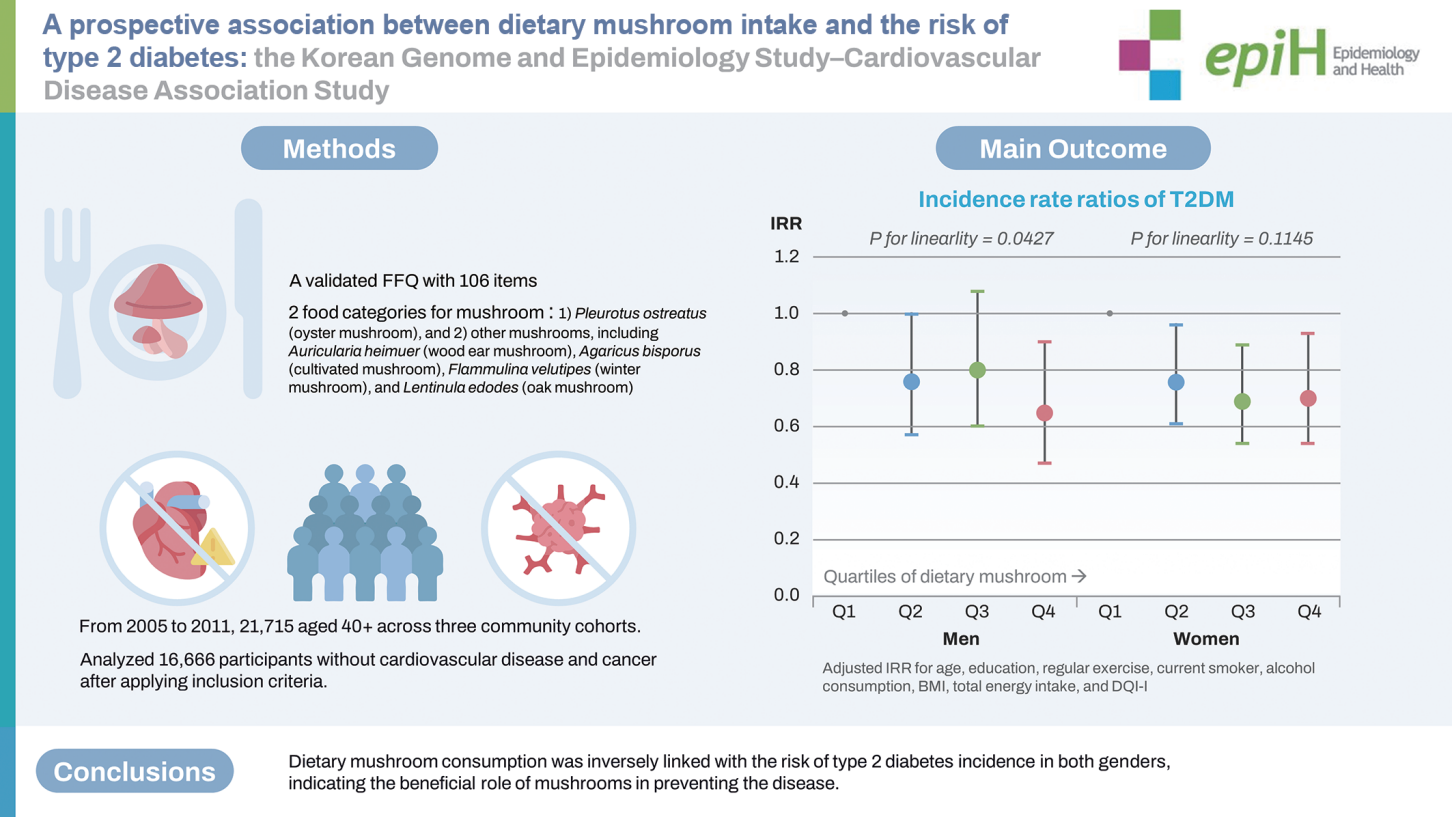


**Table 1.** Baseline characteristics of the study population and age-adjusted characteristics according to quartiles (Q) of dietary mushroom consumption

Characteristics	Total	Dietary mushroom consumption (serving/day)				p_difference_^1^	p_linearity_^2^
		Q1	Q2	Q3	Q4		
Men (n)	6,162	1,492	1,589	1,543	1,538		
Cases/person-years	410/35,831	102/7,460	103/9,820	111/9,353	94/9,198		
Median intake (Min-Max)	0.05 (0.00-2.36)	0 (0.00-0.01)	0.03 (0.01-0.05)	0.08 (0.05-0.13)	0.25 (0.19-2.36)		
Age (yr)	59.0±9.6	63.1±0.2^a^	59.6±0.2^b^	57.4±0.2^c^	56.3±0.2^d^	<0.001	<0.001
High school graduate^3^	37.8	29.1^a^	33.8^b^	42.8^c^	45.1^c^	<0.001	<0.001
Regular exercise^4^	21.0	14.1^a^	18.9^b^	23.9^c^	26.9^c^	<0.001	<0.001
Current smoker	35.8	40.1^a^	35.6^a^^b^	33.5^b^	34.4^b^	0.002	0.026
Current drinker	66.4	65.3	66.0	66.0	68.2	0.365	0.083
Alcohol consumption (g/day)	22.8±70.9	25.2±1.1^a^	22.5±1.0^a^^b^	20.5±1.0^b^	23.0±1.0^a^^b^	0.021	0.582
Body mass index (kg/m^2^)	24.1±3.0	23.8±0.1^a^	24.0±0.1^a^^b^	24.2±0.1^b^	24.3±0.1^b^	<0.001	<0.001
Total energy intake (kcal/day)	1,683±429	1,528±10.3^a^	1,600±9.8^b^	1,709±10.0^c^	1,891±10.1^d^	<0.001	<0.001
Modified diet quality Index-International score	67.3±6.9	63.9±0.2^a^	65.7±0.2^b^	68.5±0.2^c^	71.1±0.2^d^	<0.001	<0.001
Women (n)	10,504	2,697	2,564	2,621	2,622		
Cases/person-years	535/61,114	163/13,429	136/16,098	117/15,866	119/15,721		
Median intake (Min-Max)	0.05 (0.00-6.00)	0 (0.00-0.01)	0.03 (0.01-0.05)	0.10 (0.06-0.17)	0.30 (0.17-6.00)		
Age (yr)	57.7±9.8	63.3±0.2^a^	58.3±0.2^b^	55.6±0.2^c^	53.5±0.2^d^	<0.001	<0.001
High school graduate^3^	22.8	17.0^a^	16.7^a^	24.5^b^	33.0^c^	<0.001	<0.001
Regular exercise^4^	22.0	15.1^a^	18.9^b^	24.3^c^	30.0^d^	<0.001	<0.001
Current smoker	2.8	3.9^a^	2.1^b^	2.3^b^	2.9^a^^b^	<0.001	0.700
Current drinker	28.7	30.1	29.0	26.9	28.7	0.088	0.564
Alcohol consumption (g/day)	1.9±8.4	2.0±0.2	1.8±0.2	1.7±0.2	2.0±0.2	0.629	0.668
Body mass index (kg/m^2^)	24.4±3.2	24.5±0.1	24.5±0.1	24.4±0.1	24.4±0.1	0.783	0.588
Total energy intake (kcal/day)	1,489±405	1,354±7.5^a^	1,427±7.4^b^	1,509±7.4^c^	1,668±7.5^d^	<0.001	<0.001
Modified diet quality index-international score	68.3±7.5	63.9±0.1^a^	66.8±0.1^b^	69.6±0.1^c^	72.8±0.1^d^	<0.001	<0.001

Values are presented as mean±standard deviation in total participants and age-adjusted values in men and women, and mean±standard error for continuous variables or % for categorical variables; Mean values with different superscripts (a, b, c, d) within a row indicate significant differences among the exposure groups according to the Tukey multiple comparison test. Min, minimum; Max, maximum.

1 p-values for differences were determined by a general linear model (Tukey’s multiple comparisons).

2 p-values for linear trends were obtained by finding the median value of each quartile and treating it as a continuous variable.

3 High school graduate (≥12 years of education).

4 Regular exercise (≥3 times/wk and ≥30 min/session).

**Table 2.** Incidence rate ratios and 95% confidence intervals of type 2 diabetes incidence according to quartiles (Q) of dietary mushroom consumption

Variables	Dietary mushroom consumption (serving/day)				p_linearity_^1^	p_non-linearity_^2^
	Q1	Q2	Q3	Q4		
Men (n=6,162)						
Median intake (Min-Max)	0.00 (0.00-0.01)	0.03 (0.01-0.05)	0.08 (0.05-0.13)	0.25 (0.19-2.36)		
Cases/person-years	102/7,460	103/9,820	111/9,353	94/9,198		
Age-adjusted	1.00 (reference)	0.77 (0.59-1.02)	0.88 (0.67-1.17)	0.76 (0.57-1.02)	0.220	0.966
Multivariable model 1^3^	1.00 (reference)	0.75 (0.57-0.99)	0.80 (0.60-1.06)	0.65 (0.47-0.88)	0.031	0.877
Multivariable model 2^4^: Model 1+DQI-I	1.00 (reference)	0.76 (0.57-1.00)	0.80 (0.60-1.08)	0.65 (0.47-0.90)	0.043	0.891
Sensitivity analyses^4^						
Adjusted for nutrients^5^ related to type 2 diabetes instead of DQI-I score	1.00 (reference)	0.75 (0.57-0.99)	0.76 (0.57-1.01)	0.56 (0.40-0.79)	0.003	0.886
Adjusted for fasting blood glucose at baseline	1.00 (reference)	0.80 (0.61-1.06)	0.80 (0.60-1.07)	0.68 (0.49-0.94)	0.055	0.993
Excluding type 2 diabetes events that occurred within the first year	1.00 (reference)	0.83 (0.62-1.11)	0.87 (0.64-1.18)	0.71 (0.51-0.99)	0.046	0.719
Among non-users of multi-nutrients and functional dietary food supplements at baseline	1.00 (reference)	0.84 (0.58-1.21)	0.85 (0.57-1.28)	0.57 (0.36-0.90)	0.020	0.498
Women (n=10,504)						
Median intake (Min-Max)	0.00 (0.00-0.01)	0.03 (0.01-0.05)	0.10 (0.06-0.17)	0.30 (0.17-6.00)		
Cases/person-years	163/13,429	136/16,098	117/15,866	119/15,721		
Age-adjusted	1.00 (reference)	0.75 (0.60-0.95)	0.70 (0.54-0.89)	0.74 (0.57-0.95)	0.148	0.018
Multivariable model 1^3^	1.00 (reference)	0.77 (0.61-0.97)	0.71 (0.55-0.92)	0.73 (0.56-0.95)	0.132	0.031
Multivariable model 2^4^: model 1+DQI-I	1.00 (reference)	0.76 (0.61-0.96)	0.69 (0.54-0.89)	0.70 (0.54-0.93)	0.114	0.027
Sensitivity analyses^4^						
Adjusted for nutrients^5^ related to type 2 diabetes instead of DQI-I score	1.00 (reference)	0.77 (0.61-0.97)	0.71 (0.55-0.92)	0.72 (0.54-0.96)	0.150	0.032
Adjusted for fasting blood glucose at baseline	1.00 (reference)	0.81 (0.64-1.03)	0.79 (0.61-1.02)	0.72 (0.55-0.95)	0.073	0.140
Excluding type 2 diabetes events that occurred within the first year	1.00 (reference)	0.77 (0.61-0.97)	0.68 (0.53-0.89)	0.70 (0.53-0.92)	0.058	0.030
Among non-users of multi-nutrients and functional dietary food supplements at baseline	1.00 (reference)	0.75 (0.55-1.02)	0.75 (0.53-1.05)	0.67 (0.45-0.99)	0.132	0.145

Covariates obtained during the baseline survey were used, except for dietary factors. DQI-I, Diet Quality Index-International.

1 p-values for linear trend were obtained by imputing the median value of each quartile and treating it as a continuous variable using a modified Poisson regression with a robust error estimator.

2 Tests for non-linearity used the likelihood ratio test, comparing the model with only the linear term to the model with the linear and cubic spline terms.

3 Multivariable model 1 was adjusted for age (years), high school graduate (≥12 years of education), regular exercise (≥3 times/wk and ≥30 min/session), current smoker (yes or no), alcohol consumption (mL/day), body mass index (kg/m^2^), and total energy intake (kcal/day) in men and women.

4 Multivariable model 2 was additionally adjusted for the modified DQI-I score in multivariable model 1.

5 Energy-adjusted nutrients previously reported to be associated with type 2 diabetes include energy-adjusted dietary calcium (mg/day), folate (μg/day), isoflavones (mg/day), glycaemic load, iron (mg/day), fibre (g/day), and magnesium (mg/day).

**Table 3.** Stratified incidence rate ratios and 95% confidence intervals of type 2 diabetes according to quartiles (Q) of dietary mushroom by diabetes risk factors in men and women^1^

Variables	Dietary mushroom consumption (serving/day)				p_linearity_^2^	p_interaction_^3^
	Q1	Q2	Q3	Q4		
Men						
Age (yr)						
<65	1.00 (reference)	0.60 (0.43, 0.84)	0.61 (0.43, 0.86)	0.49 (0.33, 0.71)	0.003	0.043
≥65	1.00 (reference)	1.01 (0.63, 1.61)	1.25 (0.76, 2.09)	1.09 (0.62, 1.93)	0.082	
Education level (yr)						
<12	1.00 (reference)	0.67 (0.48, 0.93)	0.84 (0.59, 1.20)	0.61 (0.40, 0.91)	0.017	0.331
≥12	1.00 (reference)	0.99 (0.58, 1.69)	0.80 (0.47, 1.37)	0.78 (0.44, 1.40)	0.457	
Regular exercise						
No	1.00 (reference)	0.64 (0.47, 0.87)	0.79 (0.57, 1.09)	0.65 (0.45, 0.93)	0.036	0.035
Yes	1.00 (reference)	1.93 (0.88, 4.24)	1.16 (0.51, 2.64)	0.91 (0.39, 2.12)	0.268	
Current smoking status						
No	1.00 (reference)	0.87 (0.61, 1.26)	0.94 (0.63, 1.39)	0.64 (0.41, 0.99)	0.023	0.033
Yes	1.00 (reference)	0.63 (0.40, 0.97)	0.65 (0.41, 1.03)	0.70 (0.43, 1.15)	0.352	
Current drinking status						
No	1.00 (reference)	0.79 (0.47, 1.34)	0.99 (0.59, 1.67)	1.05 (0.60, 1.86)	0.753	0.042
Yes	1.00 (reference)	0.75 (0.54, 1.05)	0.73 (0.51, 1.05)	0.51 (0.34, 0.77)	0.002	
Body mass index (kg/m^2^)						
<23	1.00 (reference)	0.59 (0.34, 1.03)	0.38 (0.18, 0.78)	0.44 (0.21, 0.94)	0.068	0.094
≥23	1.00 (reference)	0.84 (0.61, 1.16)	0.97 (0.70, 1.36)	0.74 (0.51, 1.08)	0.081	
Prediabetes (mg/dL)						
Normal (FBG <100)	1.00 (reference)	0.66 (0.41, 1.06)	0.73 (0.43, 1.24)	0.47 (0.25, 0.88)	0.021	0.162
Prediabetic (FBG ≥100)	1.00(reference)	0.84 (0.60, 1.18)	0.82 (0.58, 1.17)	0.72 (0.49, 1.07)	0.147	
Diet quality index-international score						
< Median level	1.00 (reference)	0.83 (0.58, 1.20)	1.11 (0.75, 1.65)	1.13 (0.72, 1.77)	0.686	
≥ Median level	1.00 (reference)	0.66 (0.43, 1.01)	0.56 (0.37, 0.84)	0.43 (0.28, 0.66)	0.002	0.028
Women						
Age (yr)						
<65	1.00 (reference)	0.73 (0.54, 1.00)	0.64 (0.47, 0.88)	0.64 (0.46, 0.89)	0.069	0.989
≥65	1.00 (reference)	0.86 (0.60, 1.23)	0.86 (0.54, 1.38)	0.91 (0.52, 1.62)	0.651	
Education level (yr)						
<12	1.00 (reference)	0.74 (0.59, 0.95)	0.71 (0.54, 0.93)	0.70 (0.52, 0.94)	0.050	0.840
≥12	1.00 (reference)	1.07 (0.40, 2.82)	0.74 (0.29, 1.84)	0.80 (0.32, 2.02)	0.785	
Regular exercise						
No	1.00 (reference)	0.70 (0.54, 0.90)	0.67 (0.51, 0.89)	0.71 (0.52, 0.96)	0.090	0.296
Yes	1.00 (reference)	1.20 (0.66, 2.20)	0.84 (0.44, 1.61)	0.76 (0.39, 1.49)	0.297	
Current smoking status						
No	1.00 (reference)	0.74 (0.59, 0.94)	0.66 (0.51, 0.86)	0.71 (0.54, 0.94)	0.104	0.109
Yes	1.00 (reference)	1.44 (0.45, 4.63)	1.61 (0.56, 4.63)	0.29 (0.05, 2.36)	0.210	
Current drinking status						
No	1.00 (reference)	0.78 (0.60, 1.02)	0.64 (0.47, 0.86)	0.74 (0.54, 1.01)	0.218	0.341
Yes	1.00 (reference)	0.69 (0.42, 1.13)	0.85 (0.52, 1.40)	0.61 (0.34, 1.10)	0.114	
Body mass index (kg/m^2^)						
<23	1.00 (reference)	0.78 (0.43, 1.43)	1.03 (0.53, 1.98)	1.30 (0.63, 2.67)	0.109	0.672
≥23	1.00 (reference)	0.76 (0.59, 0.97)	0.64 (0.49, 0.84)	0.63 (0.47, 0.84)	0.015	
Prediabetes (mg/dL)						
Normal (FBG <100)	1.00 (reference)	1.14 (0.76, 1.70)	1.01 (0.66, 1.54)	0.82 (0.50, 1.34)	0.320	0.165
Prediabetic (FBG ≥100)	1.00 (reference)	0.67 (0.50, 0.90)	0.67 (0.48, 0.93)	0.69 (0.50, 0.97)	0.117	
Diet quality index-international score						
< Median level	1.00 (reference)	0.80 (0.60, 1.07)	0.75 (0.52, 1.09)	0.65 (0.39, 1.08)	0.057	0.823
≥ Median level	1.00 (reference)	0.80 (0.53, 1.20)	0.69 (0.47, 1.02)	0.70 (0.48, 1.04)	0.280	

Covariates obtained at the baseline survey were used, except for dietary factors. FBG, fasting blood glucose.

1 Multivariable model was adjusted for age (years), high school graduate (≥12 years of education), regular exercise (≥3 times/wk and ≥30 min/session), current smoker (yes or no), alcohol consumption (mL/day), body mass index (kg/m^2^), total energy intake (kcal/day), and the modified Diet Quality Index-International score in men and women.

2 p-values for linear trend were obtained by imputing the median value of each quartile and treating it as a continuous variable using a modified Poisson regression with a robust error estimator.

3 p-values for interaction were obtained by including the cross-product term of dietary mushroom intake and each variable in the Poisson regression model to test for multiplicative interactions.

## Data Availability

The authors did not produce the data and do not own the data. Data are available from the Korea Disease Control and Prevention Agency Institutional Data Access/Ethics Committee for researchers who meet the criteria for access to confidential data (https://nih.go.kr/ko/main/contents.do?menuNo=300566). Such researchers may access the data in the same way that the authors accessed them.

## Supplementary materials

Supplementary Material 1. Scoring criteria for the Diet Quality Index-International (DQI-I) and distribution of CAVAS participants for each component

Supplementary Material 2. Age-adjusted dietary nutrient intake, fasting blood glucose, and cases and person-years of two sensitivity analysis datasets according to quartiles (Q) of dietary mushroom consumption

Supplementary Material 3. Incidence rate ratio (IRR) and 95% confidence intervals (CI) of type 2 diabetes (T2D) incidence by dietary mushroom in the three cohorts
