# Supplementary material for: A prospective association between dietary mushroom intake and the risk of type 2 diabetes: the Korean Genome and Epidemiology Study–Cardiovascular Disease Association Study
Source: Epidemiol Health. 2024 Jan 8;46:e2024017. doi: 10.4178/epih.e2024017 (PMC11040214; doi:10.4178/epih.e2024017)
Supplement: Supplementary Material 1. — Scoring criteria for the Diet Quality Index-International (DQI-I) and distribution of CAVAS participants for each component [file epih-46-e2024017-Supplementary-1.docx]

**Supplementary Materials**

**Supplementary material 1.** Scoring criteria for the Diet Quality Index-International (DQI-I) and distribution of CAVAS participants for each component

**Supplemental material 2**. Age-adjusted dietary nutrient intake, fasting blood glucose, and cases and person-years of two sensitivity analysis datasets according to quartiles (Q) of dietary mushroom consumption

**Supplemental material 3**. Incidence rate ratio (IRR) and 95% confidence intervals (CI) of type 2 diabetes (T2D) incidence by dietary mushroom in the three cohorts

**Supplementary material 1.** Scoring criteria for the Diet Quality Index-International (DQI-I) and distribution of CAVAS participants for each component

| **Component^1^** | **Score^2^** | **Scoring criteria** | **Median (min, max) for**  **cumulative DQI-I scores** | |
| --- | --- | --- | --- | --- |
|  |  |  | **Men** | **Women** |
| **Cumulative average of total DQII^3^** | **0–100** |  | **67.5**  **(40.9, 87.6)** | **68.8**  **(40.3, 87.8)** |
|  |  |  |  |  |
| **[1] Variety** | **0–20** |  |  |  |
| Overall food group variety (meat/poultry/fish/eggs; dairy/beans; grain; fruit; vegetable) | *0–15* | ≥ 1 serving from each food group/d =15 | 10.5 (3, 15) | 10.5 (3, 15) |
|  |  | Any 1 food group missing/d = 12 |  |  |
|  |  | Any 2 food groups missing/d = 9 |  |  |
|  |  | Any 3 food groups missing/d = 6 |  |  |
|  |  | ≥ 4 food groups missing/d = 3 |  |  |
|  |  | None from any food groups = 0 |  |  |
| Within-group variety for protein source (meat, poultry, fish, dairy, beans, eggs) | *0–5* | ≥ 3 different sources/d = 5 | 1.0 (0, 5) | 1.0 (0, 5) |
|  |  | 2 different sources/d = 3 |  |  |
|  |  | From 1 source/d = 1 |  |  |
|  |  | None = 0 |  |  |
| **[2] Adequacy^4^** | ***0–40*** |  |  |  |
| Vegetable group | *0–5* | ≥ 3–5 servings/d = 5, 0 servings/d = 0 | 5.0 (0, 5) | 5.0 (0, 5) |
|  |  | ≥ 100% |  |  |
|  |  | < 100–50% |  |  |
|  |  | < 50% |  |  |
| Fruit group | *0–5* | ≥ 2–4 servings/d = 5, 0 servings/d = 0 | 1.9 (0, 5) | 2.6 (0, 5) |
|  |  | ≥ 100% |  |  |
|  |  | < 100–50% |  |  |
|  |  | < 50% |  |  |
| Grain group | *0–5* | ≥ 6–11 servings/d = 5, 0 servings/d = 0 | 2.7 (0.5, 5) | 2.6 (0.1, 4.7) |
|  |  | ≥ 100% |  |  |
|  |  | < 100–50% |  |  |
|  |  | < 50% |  |  |
| Fiber | *0–5* | ≥ 20–30 g/d = 5, 0 g/d = 0 | 3.6 (0.7, 5) | 3.5 (0.4, 5) |
|  |  | ≥ 100% |  |  |
|  |  | < 100–50% |  |  |
|  |  | < 50% |  |  |
| Protein | *0–5* | ≥ 10% energy/d = 5, 0 energy/d = 0 | 5.0 (4.1, 5) | 5.0 (3.5, 5) |
|  |  | ≥ 100% |  |  |
|  |  | < 100–50% |  |  |
|  |  | < 50% |  |  |
| Iron | *0–5* | ≥ 100% RNI/d= 5, 0 RNI /d = 0 | 4.6 (1, 5) | 4.6 (0.5, 5) |
|  |  | ≥ 100% |  |  |
|  |  | < 100–50% |  |  |
|  |  | < 50% |  |  |
| Calcium | *0–5* | ≥ 100% RNI /d= 5, 0 RNI /d = 0 | 2.2 (0.2, 5) | 2.0 (0.1, 5) |
|  |  | ≥ 100% |  |  |
|  |  | < 100–50% |  |  |
|  |  | < 50% |  |  |
| Vitamin C | *0–5* | ≥ 100% RNI /d= 5, 0 RNI /d = 0 | 3.3 (0, 5) | 3.5 (0, 5) |
|  |  | ≥ 100% |  |  |
|  |  | < 100–50% |  |  |
|  |  | < 50% |  |  |
| **[3] Moderation** | ***0–30*** |  |  |  |
| Total fat | *0–6* | ≤ 20 % of total energy/d = 6 | 6 (0, 6) | 6 (0, 6) |
|  |  | > 20–30 % of total energy/d = 3 |  |  |
|  |  | > 30 % of total energy/d = 0 |  |  |
| Saturated fat | *0–6* | ≤ 7 % of total energy/d = 6 | 6 (0, 6) | 6 (0, 6) |
|  |  | > 7–10 % of total energy/d = 3 |  |  |
|  |  | > 10 % of total energy/d = 0 |  |  |
| Cholesterol | *0–6* | ≤ 300 mg/d = 6 | 6 (0, 6) | 6 (0, 6) |
|  |  | > 300–400 mg/d = 3 |  |  |
|  |  | > 400 mg/d = 0 |  |  |
| Sodium | *0–6* | ≤ 2400 mg/d = 6 | 3 (0, 6) | 4.5 (0, 6) |
|  |  | > 2400–3400 mg/d = 3 |  |  |
|  |  | > 3400 mg/d = 0 |  |  |
| Empty calorie foods | *0–6* | ≤ 3 % of total energy/d = 6 | 6 (0, 6) | 6 (0, 6) |
|  |  | > 3–10 % of total energy/d = 3 |  |  |
|  |  | > 10 % of total energy/d = 0 |  |  |
| **[4] Overall balance** | ***0–10*** |  |  |  |
| Macronutrient ratio (carbohydrate:protein: fat) | *0–6* | 5 –65 : 10–15 : 15–25 = 6 | 0 (0, 5) | 0 (0, 4) |
|  |  | 52–68 : 9–16 : 13–27 = 4 |  |  |
|  |  | 50–70 : 8–12 : 12–30 = 2 |  |  |
|  |  | Otherwise = 0 |  |  |
| Fatty acid ratio (PUFA:MUFA:SFA) | *0–4* | P/S 1–1.5 and M/S 1–1.5 = 4 | 0 (0, 4) | 0 (0, 4) |
|  |  | Else if P/S 0.8–1.7 and M/S 0.8–1.7 = 2 |  |  |
|  |  | Otherwise = 0 |  |  |

^1^ Reference: Kim, S., Haines, P. S., Siega-Riz, A. M., & Popkin, B. M. (2003). The Diet Quality Index-International (DQI-I) provides an effective tool for cross-national comparison of diet quality as illustrated by China and the United States. The Journal of nutrition, 133(11), 3476-3484.

^2^ Scores for each component are summarized in each of the four main categories, and the scores for all four categories are summed, resulting in the total DQI-I score, ranging from 0 to 100 (0 being the poorest and 100 being the highest possible score)

^3^ Cumulative average = Σ(i=1 to 3) (Total DQII score summed for each component of the i-th dietary data / Number of dietary data utilized with censoring considered)

^4^ The Adequacy scores for the eight components in the category are assigned on the basis of the percentage attainment of the recommended intakes on a continuous scale, which ranges from 0 points for 0% to 5 points for 100%, with a cap at 5 points.
