# Supplementary material for: A prospective association between dietary mushroom intake and the risk of type 2 diabetes: the Korean Genome and Epidemiology Study–Cardiovascular Disease Association Study
Source: Epidemiol Health. 2024 Jan 8;46:e2024017. doi: 10.4178/epih.e2024017 (PMC11040214; doi:10.4178/epih.e2024017)
Supplement: Supplementary Material 2. — Age-adjusted dietary nutrient intake, fasting blood glucose, and cases and person-years of two sensitivity analysis datasets according to quartiles (Q) of dietary mushroom consumption [file epih-46-e2024017-Supplementary-2.docx]

**Supplemental material 2**. Age-adjusted dietary nutrient intake, fasting blood glucose, and cases and person-years of two sensitivity analysis datasets according to quartiles (Q) of dietary mushroom consumption

| Characteristics | Total | Dietary mushroom consumption (serving/d) | | | | *P* _difference_^3^ | *P* _linearity_^4^ |
| --- | --- | --- | --- | --- | --- | --- | --- |
|  |  | Q1 | Q2 | Q3 | Q4 |  |  |
| **MEN** |  |  |  |  |  |  |  |
| *N* | ***6,162*** | ***1,492*** | ***1,589*** | ***1,543*** | ***1,538*** |  |  |
| Cases/Person years | 410 / 35,831 | 102 / 7,460 | 103 / 9,820 | 111 / 9,353 | 94 / 9,198 |  |  |
| Median intake (min, max) | 0.05 (0, 2.36) | 0 (0, 0.01) | 0.03 (0.01, 0.05) | 0.08 (0.05, 0.13) | 0.25 (0.19, 2.36) |  |  |
|  |  |  |  |  |  |  |  |
| **Age-adjusted dietary intake of** |  |  |  |  |  |  |  |
| Calcium (mg/d) | 340.7 ± 132 | 311.6 ± 3.4 ^a^ | 315.4 ± 3.2 ^a^ | 348.4 ± 3.3 ^b^ | 387.5 ± 3.3 ^c^ | <.0001 | <.0001 |
| Folate (μg/d) | 401.9 ± 118.3 | 383.6 ± 3.0 ^a^ | 377.1 ± 2.9 ^a^ | 398.5 ± 2.9 ^b^ | 448.5 ± 3.0 ^c^ | <.0001 | <.0001 |
| Isoflavones (mg/d) | 17.3 ± 12.8 | 15.8 ± 0.3 ^a^ | 16.3 ± 0.3 ^a^ | 17.9 ± 0.3 ^b^ | 19.1 ± 0.3 ^b^ | <.0001 | <.0001 |
| Glycemic load | 188.5 ± 18.8 | 193.1 ± 0.5^a^ | 191.8 ± 0.5 ^a^ | 187.2 ± 0.5 ^b^ | 182.1 ± 0.5 ^c^ | <.0001 | <.0001 |
| Iron (mg/d) | 9.6 ± 2.0 | 9.0 ± 0.1 ^a^ | 9.1 ± 0.1 ^a^ | 9.7 ± 0.1 ^b^ | 10.7 ± 0.1 ^c^ | <.0001 | <.0001 |
| Fiber (g/d) | 15.5 ± 4.5 | 14.6 ± 0.1 ^a^ | 14.6 ± 0.1 ^a^ | 15.4 ± 0.1 ^b^ | 17.2 ± 0.1 ^c^ | <.0001 | <.0001 |
| Magnesium (mg/d) | 95.3 ± 17.5 | 94.1 ± 0.5 ^a^ | 93.1 ± 0.4 ^a^ | 94.1 ± 0.4 ^a^ | 100.1 ± 0.4 ^b^ | <.0001 | <.0001 |
| **Fasting blood glucose at baseline** | 95.5 ± 10.1 | 95.4 ± 0.27 | 95.4 ± 0.25 | 95.6± 0.26 | 95.7 ± 0.26 | 0.8430 | 0.4005 |
|  |  |  |  |  |  |  |  |
| **Cases/person-years** |  |  |  |  |  |  |  |
| After excluding cases of type 2 diabetes that occurred within the first year | 318/35,819 | 89 / 7,453 | 99 / 9,819 | 105 / 9,352 | 88 / 9,195 |  |  |
| Among non-users of multinutrients and functional dietary food supplements at baseline | 206/17,694 | 59 / 4,670 | 58 / 5,094 | 56 / 4,367 | 33 / 3,563 |  |  |
|  |  |  |  |  |  |  |  |
| **WOMEN** |  |  |  |  |  |  |  |
| *N* | ***10,504*** | ***2,697*** | ***2,564*** | ***2,621*** | ***2,622*** |  |  |
| Cases/Person-years | 535 /61,114 | 163 / 13,429 | 136 / 16,098 | 117 / 15,866 | 119 / 15,721 |  |  |
| Median intake (min, max) | 0.05 (0, 6.00) | 0 (0, 0.01) | 0.03 (0.01, 0.05) | 0.10 (0.06, 0.17) | 0.30 (0.17, 6.00) |  |  |
|  |  |  |  |  |  |  |  |
| **Age--adjusted dietary intake of** |  |  |  |  |  |  |  |
| Calcium (mg/d) | 341.9 ± 144.9 | 289.8 ± 2.8 ^a^ | 318.0 ± 2.7 ^b^ | 353.8 ± 2.7 ^c^ | 407.1 ± 2.8 ^d^ | <.0001 | <.0001 |
| Folate (μg/d) | 380.9 ± 118.4 | 346.6 ± 2.3 ^a^ | 359.0 ± 2.2 ^b^ | 386.1 ± 2.2 ^c^ | 432.3 ± 2.3 ^d^ | <.0001 | <.0001 |
| Isoflavones (mg/d) | 16.6 ± 12.4 | 14.3 ± 0.2 ^a^ | 16.0 ± 0.2 ^b^ | 16.7 ± 0.2 ^b^ | 19.6 ± 0.2 ^c^ | <.0001 | <.0001 |
| Glycemic load | 170.6 ± 18.8 | 178.1 ± 0.3 ^a^ | 173.6 ± 0.3 ^b^ | 168.5 ± 0.3 ^c^ | 162.1 ± 0.3 ^d^ | <.0001 | <.0001 |
| Iron (mg/d) | 9.1 ± 2.2 | 8.2 ± 0.04 ^a^ | 8.6 ± 0.04 ^b^ | 9.2 ± 0.04 ^c^ | 10.3 ± 0.04 ^d^ | <.0001 | <.0001 |
| Fiber (g/d) | 14.7 ± 4.5 | 13.2 ± 0.1 ^a^ | 13.8 ± 0.1 ^b^ | 14.8 ± 0.1 ^c^ | 16.7 ± 0.1 ^d^ | <.0001 | <.0001 |
| Magnesium (mg/d) | 85.9 ± 17.4 | 83.7 ± 0.3 ^a^ | 82.8 ± 0.3 ^a^ | 85.6 ± 0.3 ^b^ | 91.7 ± 0.3 ^c^ | <.0001 | <.0001 |
| **Fasting blood glucose at baseline** | 92.4 ± 9.4 | 92.9 ± 0.19 ^a^ | 92.1 ± 0.19 ^b^ | 92.1 ± 0.18 ^b^ | 92.4 ± 0.19 ^b^ | 0.0046 | 0.5610 |
|  |  |  |  |  |  |  |  |
| **Cases/person-years** |  |  |  |  |  |  |  |
| After excluding cases of type 2 diabetes that occurred within the first year | 506/61,102 | 153 / 13,426 | 130 / 16,095 | 110 / 15,864 | 113 / 15,717 |  |  |
| Among non-users of multi-nutrients and functional dietary food supplements at baseline | 281/30,368 | 107 / 8,813 | 72 / 8,509 | 58 / 7,075 | 44 / 5,971 |  |  |

The values are expressed as mean ± SD in total participants and age-adjusted values in men and women, and mean ± SE for continuous variables or percentage for categorical variables. All nutrients were energy-adjusted values using the residual method. Covariates obtained at the baseline survey were used, except for dietary factors.

^1^ High school graduate (≥ 12 years of education).

^2^ Regular exercise (≥ 3 times/week and ≥ 30 min/session)

^3^ *P* values for differences were determined by general linear model (Tukey’s multiple comparisons)

^4^ *P* values for linear trends were obtained by the median value of each quartile and treating it as a continuous variable.
