# Supplementary material for: A prospective association between dietary mushroom intake and the risk of type 2 diabetes: the Korean Genome and Epidemiology Study–Cardiovascular Disease Association Study
Source: Epidemiol Health. 2024 Jan 8;46:e2024017. doi: 10.4178/epih.e2024017 (PMC11040214; doi:10.4178/epih.e2024017)
Supplement: Supplementary Material 3. — Incidence rate ratio (IRR) and 95% confidence intervals (CI) of type 2 diabetes (T2D) incidence by dietary mushroom in the three cohorts [file epih-46-e2024017-Supplementary-3.docx]

Supplemental **material** 3. Incidence rate ratio (IRR) and 95% confidence intervals (CI) of type 2 diabetes (T2D) incidence by dietary mushroom in the three cohorts

|  | Dietary mushroom consumption (serving/d) | | | | | | | | | |
| --- | --- | --- | --- | --- | --- | --- | --- | --- | --- | --- |
|  | MEN | | | | *P* _linearity_ ^2^ | WOMEN | | | | *P* _linearity_ ^2^ |
|  | Q1 | Q2 | Q3 | Q4 |  | Q1 | Q2 | Q3 | Q4 |  |
| **MRCohort** |  |  |  |  |  |  |  |  |  |  |
| Median intake (min-max, serving/d) | 0 (0-0.01) | 0.03 (0.01-0.05) | 0.08 (0.05-0.18) | 0.24 (0.13-2.0) |  | 0 (0-0.01) | 0.03 (0.01-0.05) | 0.09 (0.06-1.67) | 0.29 (0.17-3.5) |  |
| No. of cases / person years | 59 / 4,703 | 61 / 5,239 | 54 / 4,504 | 37 / 3,713 |  | 188 / 9,261 | 87 / 9,213 | 68 / 7,405 | 49 / 6,249 |  |
| Multivariable model^1^ | 1.00 | 0.84 (0.58-1.20) | 0.76 (0.51-1.13) | 0.57 (0.36-0.90) | 0.0229 | 1.00 | 0.78 (0.59-1.03) | 0.78 (0.57-1.07) | 0.66 (0.45-0.95) | 0.0743 |
| **ARIRANG** |  |  |  |  |  |  |  |  |  |  |
| Median intake (min-max, serving/d) | 0 (0-0.01) | 0.03 (0.01-0.05) | 0.08 (0.05-0.13) | 0.25 (0.13-2.0) |  | 0 (0-0.01) | 0.03 (0.01-0.05) | 0.10 (0.05-0.17) | 0.32 (0.17-4.0) |  |
| No. of cases / person years | 26 / 1,597 | 20 / 2,874 | 35 / 3,095 | 37 / 3,645 |  | 22 / 2,414 | 32 / 3,943 | 29 / 5,616 | 51 / 6,258 |  |
| Multivariable model | 1.00 | 0.44 (0.24-0.78) | 0.73 (0.43-1.24) | 0.59 (0.33-1.06) | 0.5523 | 1.00 | 1.00 (0.59-1.71) | 0.64 (0.36-1.13) | 0.98 (0.56-1.71) | 0.6253 |
| **Kangwha** |  |  |  |  |  |  |  |  |  |  |
| Median intake (min-max, serving/d) | 0 (0-0.01) | 0.03 (0.01-0.05) | 0.08 (0.05-0.13) | 0.25 (0.13-2.36) |  | 0 (0-0.01) | 0.03 (0.01-0.05) | 0.10 (0.06-0.17) | 0.34 (0.17-6.0) |  |
| No. of cases / person years | 17 / 1,160 | 22 / 1,707 | 22 / 1,754 | 20 / 1,840 |  | 23 / 1754 | 17 / 2,943 | 20 / 2,845 | 19 / 3,214 |  |
| Multivariable model | 1.00 | 0.94 (0.49-1.80) | 1.02 (0.51-2.05) | 0.98 (0.44-2.15) | 0.9974 | 1.00 | 0.48 (0.25-0.90) | 0.60 (0.31-1.15) | 0.50 (0.45-1.02) | 0.2922 |
|  |  |  |  |  |  |  |  |  |  |  |
| **Pooled-analysis** ^3^ |  |  |  |  |  |  |  |  |  |  |
| Multivariable model | 1.00 | 0.74 (0.56-0.97) | 0.79 (0.59-1.06) | 0.63 (0.46-0.88) | 0.6765 | 1.00 | 0.76 (0.61-0.97) | 0.72 (0.56-0.93) | 0.70 (0.52-0.93) | 0.0871 |

Covariates obtained at the baseline survey were used, except for dietary factors.

^1^ Multivariable model was adjusted for age (years), high school graduate (≥ 12 years of education), regular exercise (≥ 3 times/week and ≥ 30 minutes/session), current smoker (yes or no), alcohol consumption (ml/d), Body Mass Index (kg/m^2^), total energy intake (kcal/d), and modified Diet Quality Index-International (DQI-I) score in men and women.

^2^ *P* for linear trend were obtained by imputing the median value of each quartile and treating it as a continuous variable using a modified Poisson regression with a robust error estimator.

^3^ The pooled IRRs in the multivariable-adjusted models across the 3 cohorts were combined using an inverse variance-weighted fixed-effects meta-analysis.
